# Analysis of granulysin expression in vitiligo and halo-nevus

**DOI:** 10.1038/s41598-024-67494-9

**Published:** 2024-07-17

**Authors:** Nika Hlača, Marijana Vičić, Marija Kaštelan, Andrea Dekanić, Larisa Prpić-Massari

**Affiliations:** 1 Department of Dermatovenerology, Faculty of Medicine, University of Rijeka, Clinical Hospital Center Rijeka, Krešimirova 42, 51000 Rijeka, Croatia; 2 Department of Pathology, Faculty of Medicine, University of Rijeka, Clinical Hospital Center Rijeka, Krešimirova 42, 51000 Rijeka, Croatia

**Keywords:** CD8+ T cells, Granulysin, Halo-nevus, Melanocytes, Natural killer cells, Vitiligo, Vitiligo, T cells

## Abstract

Vitiligo and halo nevus are immune-mediated skin diseases that have a similar pathogenesis and involve cellular cytotoxicity mechanisms that are not yet fully understood. In this study, we investigated the expression patterns of the cytolytic molecule granulysin (GNLY) in different cytotoxic cells in skin samples of vitiligo and halo nevus. Skin biopsies were taken from perilesional and lesional skin of ten vitiligo patients, eight patients with halo nevus and ten healthy controls. We analysed the expression of GNLY by immunohistochemistry in CD8+ and CD56+ NK cells. A significantly higher accumulation of GNLY+, CD8+ GNLY+ and fewer CD56+ GNLY+ cells was found in the lesional skin of vitiligo and halo nevus than in the healthy skin. These cells were localised in the basal epidermis and papillary dermis, suggesting that GNLY may be involved in the immune response against melanocytes. Similarly, but to a lesser extent, upregulation of GNLY+ and CD8+ GNLY+ cells was observed in the perilesional skin of vitiligo and halo nevus compared to healthy controls. In this study, we demonstrated for the first time an increased expression of CD8+ GNLY+ T lymphocytes and CD56+ GNLY+ NK cells in lesions of vitiligo and halo nevus, indicating the role of GNLY in the pathogenesis of both diseases.

## Introduction

Vitiligo and halo nevus are immune-mediated skin disorders in which autoreactive CD8+ T lymphocytes attack melanocytes, resulting in well-demarcated depigmented skin patches^1–4^. Some authors consider halo nevus to be a subtype of vitiligo and even a risk factor for the progression of vitiligo, as multiple halo nevi are a marker for a cellular immune response to nested melanocytes^5,6^. Although the pathogenesis of vitiligo and halo nevus is still not completely understood, both are multifactorial diseases caused by an interplay of genetic and environmental factors that subsequently trigger an immune response against melanocytes^3,7,8^. Following exposure to environmental stressors, melanocytes release reactive oxygen species (ROS), which have the potential to alter cellular DNA, proteins and lipids^9–11^. This leads to the production of various damage-associated molecular patterns (DAMPs) such as melanocyte-specific antigens, miRNAs and heat shock proteins (HSP), which stimulate IFN-γ production and the production of CXCL9 and CXCL10, leading to the recruitment of autoreactive CD8+ T cells in the skin via their common C-X-C chemokine receptor 3 (CXCR3)^7,12–16^.

It is already known that cytotoxic CD8+ cells play a central role in the development and clinical course of vitiligo and halo nevus^3,17–19^. It has also been shown that melanocyte apoptosis is mediated by granzyme B and perforin released by CD8+ T lymphocytes, or alternatively by chemokine induction of the CXCR3B receptor on melanocytes^20,21^. Generally, CD8+ T lymphocytes and NK cells exert cytotoxic effects on target cells via two different pathways: either by secretion of granzyme B, perforin and granulysin (GNLY) or by binding of ligands and death receptors, namely FasL/Fas and TRAIL/TRAIL-R1/R2^21–24^. In addition to perforin and granzyme B, significant upregulation of FasL has recently been demonstrated in the lesional and perilesional skin of vitiligo patients, further confirming the role of adaptive cell cytotoxicity in the pathogenesis of vitiligo^21^. However, data on the role of GNLY-mediated cytotoxicity in the development of vitiligo and halo nevi are currently lacking.

Granulysin is a cytolytic, pore-forming molecule that is stored in the granules of T lymphocytes and NK cells^24,25^. By binding to the membrane of the target cells, granulysin leads to pore formation and apoptosis. It also acts synergistically with perforin to induce cell apoptosis^24,25^. Granulysin is expressed in some innate cells (NK, gamma delta T lymphocytes, NKT cells) and in CD4+ and CD8+ T lymphocytes^24^. The cytotoxic effect of GNLY has already been demonstrated in tumours and cells infected with a variety of microorganisms^26,27^. In addition, GNLY also has a proinflammatory effect and acts as a chemoattractant for T lymphocytes, monocytes, NK cells, and DCs^25^. Consequently, GNLY is involved in the development of numerous immune-mediated diseases such as graft-versus-host disease, type I diabetes and multiple sclerosis as well as some immune-mediated skin diseases, such as psoriasis, alopecia areata, lichen planus and Stevens-Johnson syndrome/toxic epidermal necrolysis (SJS/TEN)^28–32^. Therefore, it would be important to clarify the role of GNLY in the development of vitiligo and halo nevus, which has not been investigated so far. To this end, we investigated the expression of GNLY in the lesional and perilesional skin of vitiligo and halo nevus, its localisation in different skin compartments and colocalisation with CD8+ and CD56+ antigens to identify the cells expressing this cytotoxic molecule and the potential sites of immune response.

## Materials and methods

### Patients

Ten patients with clinically defined and histopathologically confirmed vitiligo (age 45–68 years, mean age 55 years) and eight patients with halo nevus (age 20–44 years, mean age 32 years) were recruited for the study after informed consent had been obtained. The control group consisted of 10 healthy volunteers (age 33–68 years, mean age 54 years) who were matched in age and gender to the groups studied. The study included seven men and three women in the vitiligo group, five women and three men in the halo nevus group and six men and four women in the healthy control (HC) group. Disease activity and severity in vitiligo patients was assessed using the Vitiligo Extent Score (VES) and the Vitiligo Disease Activity (VIDA) score^33^. The VES score ranged between 1.8–14.6 (mean VES 4.79) and the VIDA score between 0 and + 4 (mean VIDA 2.4). The VIDA score was categorised as follows + 4: new lesions in the last six weeks or less, + 3: new lesions in the last 6 weeks to 3 months; + 2: new lesions in the last 3 to 6 months; +1: new lesions in the last 6 to 12 months; 0: stable for one year or more; -1: stable for one year or more with spontaneous repigmentation. None of the patients had received systemic or topical therapy for at least 4 weeks prior to the biopsy. The ethics committee of the Clinical Hospital Centre Rijeka, Faculty of Medicine, University of Rijeka, approved the study. Informed consent was obtained from each patient and the examinations were conducted in accordance with the principles of the Declaration of Helsinki.

### Skin biopsies

Punch biopsies with a diameter of 5 mm each were taken from the lesional and perilesional skin of each vitiligo patient under local anaesthesia. The perilesional skin biopsy was taken from normal-appearing skin, 5 mm from the outer edge of the vitiligo lesions. The biopsies were taken from trunk (n = 6), upper and lower extremities (n = 3) and wrist area (n = 1). Some of the lesions showed clinical signs of active disease such as trichrome lesions (n = 2), confetti-like depigmentation (n = 3) and inflammatory vitiligo (n = 1), while others appeared clinically stable (n = 4). The halo nevi were completely removed to analyse the lesional and perilesional skin. Skin samples from healthy controls were taken from the periphery of surgical excisions of benign tumours, namely fibromas and seborrhoeic keratoses. The tissue samples were fixed in buffered formalin, embedded in paraffin and used for histopathological diagnosis confirmation and immunohistochemical analysis.

### Immunohistochemistry

Paraffin-embedded tissue was cut into 3 μm sections, deparaffinised and rehydrated through graded alcohols. Antigen retrieval was performed by microwave treatment in Tris/EDTA buffer at pH 9 for 15 min, followed by cooling at 22 °C (room temperature; RT) for 20 min. Slides were blocked with 5% BSA before incubation with mouse monoclonal anti-GNLY antibody (Leica Biosystems, Novocastra, UK) or antibody diluent (DAKO, Carpenteria, CA, USA) for 1 h at RT. The dilution ratio was 1:20 and specific binding was detected using the EnVision/DAB+ system (DAKO, Glostrup, Denmark). Tris-buffered saline (TBS) was used to wash the slides between each step. A diaminobenzidine (DAB) was distilled onto the slides and left for 10 min. Slides were then washed and incubated for 30 min with a mouse monoclonal anti-CD8 antibody (clone C8/144B) (diluted 1:100) or a mouse monoclonal anti-CD56 antibody (clone MRQ-42) (diluted 1:1000) (both DAKO, Glostrup, Denmark) for 30 min at RT, as previously described^30,31^. An irrelevant mouse IgG monoclonal antibody was used as a negative control. After washing, biotinylated secondary goat anti-mouse antibodies were added for 30 min, followed by streptavidin with alkaline phosphatase for 30 min (DAKO Real Detection System Alkaline Phosphatase/RED Rabbit Mouse) at RT. Tris-buffered saline (TBS) was used to wash the slides between each step, as previously described^30,31^. The sections were counterstained with haematoxylin. The positive cells were counted in 10 fields with representative inflammatory infiltrates in both the epidermis and dermis under 400 × magnification by two independent examiners. Single immunohistochemistry results were calculated as the percentage of positive immunolabelled cells out of the total number of cells in each selected area. Double immunohistochemistry results were calculated as the percentage of double positively stained CD8+ GNLY+ cells among all CD8+ T lymphocytes and double positively stained CD56+ GNLY+ cells among all CD56+ NK cells in each selected area. The percentage of epidermal positive cells was added to the percentage of dermal positive cells to obtain the sum of positive immunolabelled cells in the lesions and perilesional skin of vitiligo and halo nevus.

### Statistical analysis

The results were analysed using Statistica 13.2 data analysis software (StatSoft, Inc., Tulsa, OK, USA) and JASP 0.18.3 computer software. The differences between the groups were assessed using the Mann–Whitney U test and the Kruskal–Wallis test. Statistical significance was set at *p* < 0.05. Data are presented as median values and as 25/75% values (25th percentile/75th percentile). For the presentation of our data, we chose the box-whisker diagram to visualise our data by quartiles, with the whiskers indicating the variability outside the upper and lower quartiles (25/75%). The correlation analyses were calculated using the Spearman rank correlation equation. p-values below 0.05 were considered statistically significant.

### Institutional review board statement

The study was conducted in accordance with the Declaration of Helsinki and approved by the Ethics Committee of Clinical Hospital Centre Rijeka, Medical Faculty University of Rijeka (protocol code 2170-29-02/1–20-2 and date 19.12.2020).

### Informed consent

Informed consent was obtained from all subjects involved in the study.

## Results

### GNLY+ cells, CD8+ T lymphocytes and CD56+ NK cells are upregulated in lesions of vitiligo and halo nevus

**Figure 1:**
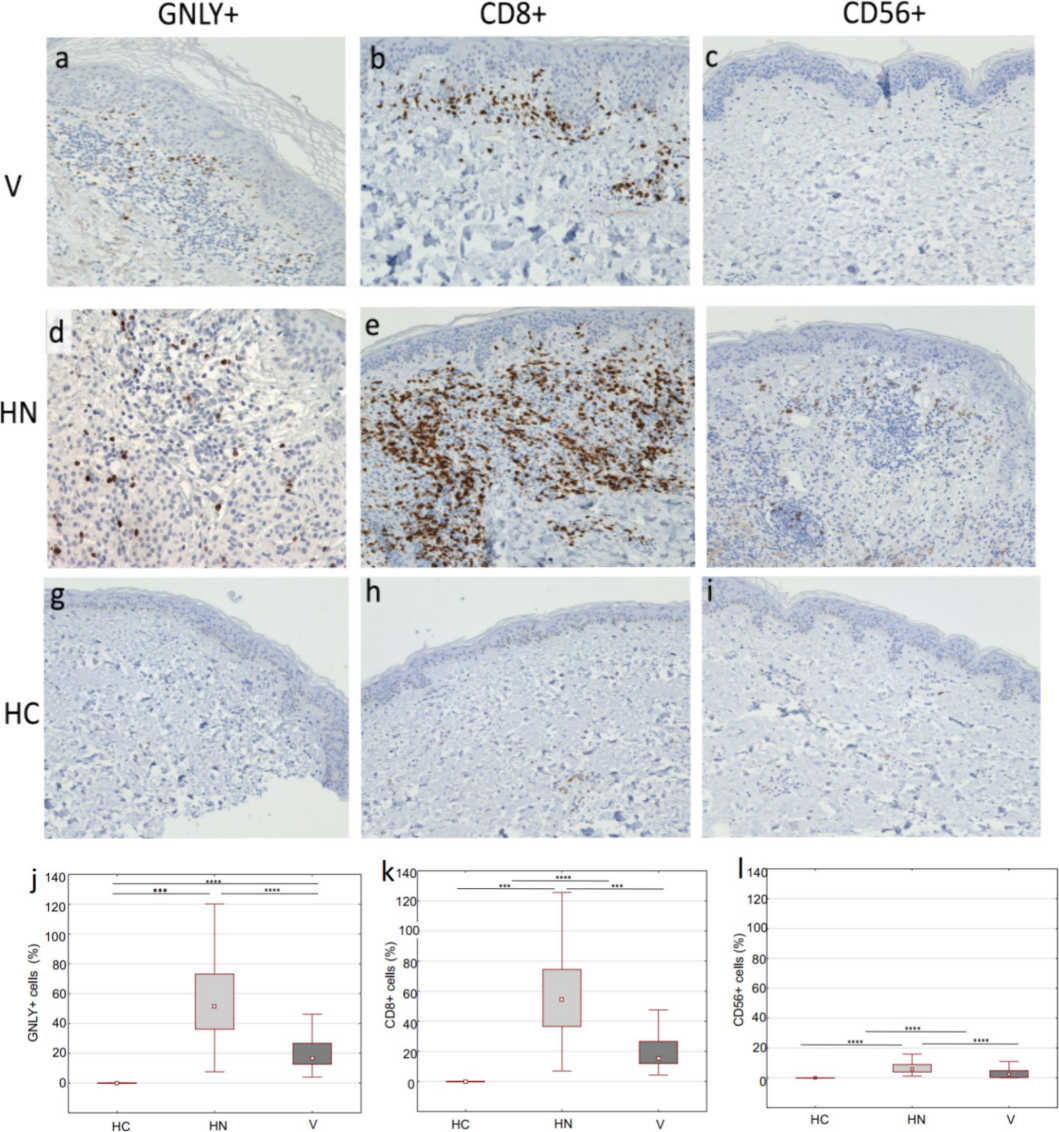
The skin lesions in vitiligo and halo nevus are infiltrated with GNLY+, CD8+ T cells and fewer CD56+ cells. Immunohistochemical staining of GNLY+, CD8+ T and CD56 cells in lesional skin biopsies of vitiligo, halo nevus and HC (**a**–**i**; magnification × 200). Quantitative analysis of cells expressing GNLY, CD8 and CD56 after immunohistochemical staining in patients with vitiligo (n = 10), halo nevus (n = 8) and HC (n = 10) (**j**–**l**). Results are expressed as the median value-25th/75th percentile. Level of significance: ****p < 0.0001, ***p < 0.001, **p < 0.01. *HC* healthy controls, *V* vitiligo, *HN* halo nevus.

The expression of GNLY+ cells was significantly higher in lesional skin of vitiligo and halo nevus compared to healthy skin, with significantly higher expression in halo nevus compared to vitiligo lesions (Fig. 1j). GNLY+ cells were mainly accumulated in the dermal compartments of vitiligo lesions and less in the basal and suprabasal compartments of the epidermis (Fig. 1a). In halo nevus, GNLY+ cells were found in the epidermis and dermis surrounding nests of nevus cells (Fig. 1d). On the other hand, in healthy controls (HC), GNLY+ cells were completely absent (Fig. 1g). A significant accumulation of CD8+ cells was found in the lesional skin of vitiligo and halo nevus compared to HC (Fig. 1k), however with a significantly higher expression in halo nevus than in vitiligo lesions. In vitiligo, CD8+ T lymphocytes are mainly found in the papillary dermis and to a lesser extent in the basal epidermis (Fig. 1b), whereas in halo nevus lesions CD8+ cells were accumulated throughout the dermis and along the dermo-epidermal junction (Fig. 1e). Less dense infiltrates of CD56+ NK cells are seen in lesions of vitiligo (Fig. 1c) and halo nevus (Fig. 1f), yet they are significantly higher in lesions of vitiligo and halo nevus than in HC (Fig. 1i, lh).

### Increased expression of GNLY+ cells, CD8+ T lymphocytes and CD56+ NK cells in the perilesional skin of vitiligo and halo nevus

**Figure 2:**
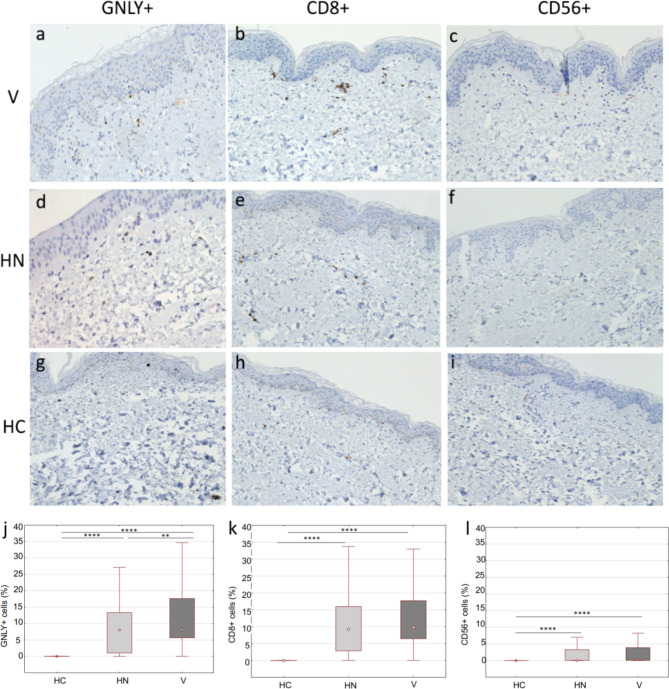
Immunohistochemical staining of GNLY+, CD8+ T and CD56+ cells in perilesional skin biopsies of vitiligo, halo nevus and HC (**a**–**i**; magnification × 200). Quantitative analysis of cells expressing GNLY, CD8 and CD56 after immunohistochemical staining in patients with vitiligo (n = 10), halo nevus (n = 8) and HC (n = 10) (**j**–**l**). Results are expressed as the median value-25th/75th percentile. Level of significance: ****p < 0.0001, ***p < 0.001, **p < 0.01. *HC* healthy controls, *V* vitiligo, *HN* halo nevus.

GNLY+ cells are significantly more abundant in the perilesional skin of vitiligo and halo nevus compared to HC (Fig. 2j). This upregulation of GNLY+ cells was found in the epidermal and dermal compartments of perilesional skin in both diseases (Fig. 2a, d). Similar to lesional skin in vitiligo, distribution analysis showed that GNLY+ cells were mainly found in dermal infiltrates and in the basal layer of the epidermis (Fig. 2a). In halo nevus, however, we observed an increased expression of GNLY+ cells in the papillary dermis (Fig. 2d). Positive cells were completely absent in healthy skin (Fig. 2g). When we compared lesional and perilesional skin, GNLY+ cells were significantly more abundant in the lesions than in the perilesional skin and in HC, in both diseases.

Increased expression of CD8+ T lymphocytes (Fig. 2b, e, k), and CD56+ NK cells (Fig. 2c, f, l) was observed in the perilesional skin of both, vitiligo and halo nevus, compared to HC. Remarkably, significantly more GNLY+ cells were present in the perilesional skin of vitiligo than in halo nevus (Fig. 2j). In addition, more CD8+ T cells and NK cells were present in the perilesional skin of vitiligo than in halo nevus, although this difference was not statistically significant. In contrast, we found no positive cells in HC (Fig. 2h, i). In addition, the expression of CD8+ T lymphocytes and CD56+ NK cells was higher in the lesions of vitiligo and halo nevus than in the perilesional skin in both diseases.

### CD8+ T lymphocytes, but not CD56+ NK cells expressing GNLY, predominate in lesions of vitiligo and halo nevus

**Figure 3:**
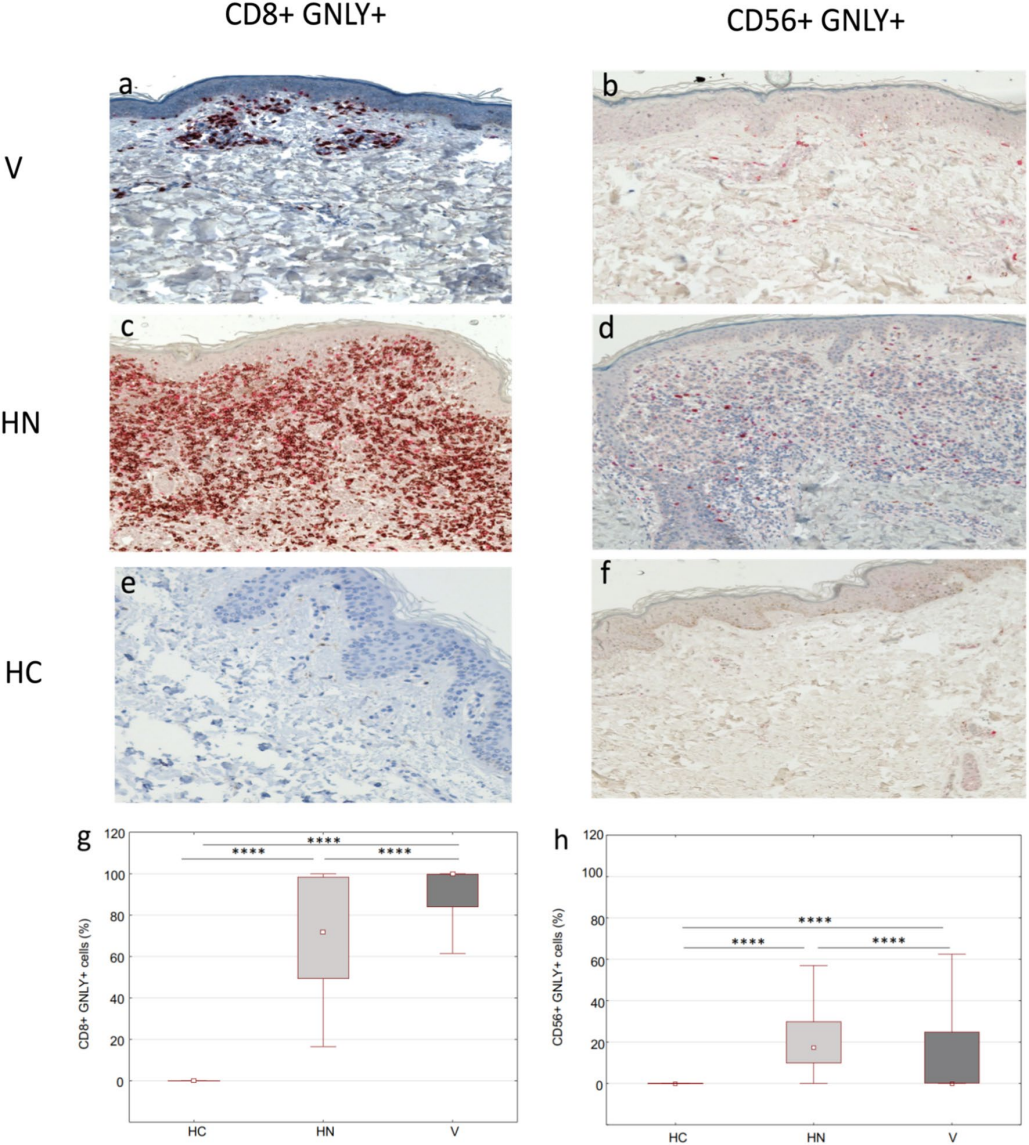
Immunohistochemical evaluation of double­positive GNLY+ CD8+ and GNLY+ CD56+ cells in lesions of vitiligo and halo nevus compared to HC (**a**–**f**; magnification × 200). Surface markers CD8 and CD56 are stained red and GNLY is stained brown. Double-positive CD8+ GNLY+ cells and CD56+ GNLY+ cells are stained with both colours. Quantitative analysis of double­positive GNLY+ CD8+ and GNLY+ CD56+ cells in lesions of vitiligo (n = 10) and halo nevus (n = 8) compared to HC (n = 10) (**g**, **h**). Results are expressed as the median value-25th/75th percentile. Level of significance: ****p < 0.0001, ***p < 0.001, **p < 0.01. *HC* healthy controls, *V* vitiligo, *HN* halo nevus.

A significantly higher accumulation of CD8+ GNLY+ cells was found in the lesional skin of vitiligo and halo nevus compared to HC (Fig. 3g). The majority of CD8+ GNLY+ cells were located in the dermis and to a lesser extent in the basal epidermis of vitiligo (Fig. 3a), whereas they were completely absent in healthy skin. In halo nevus, we detected a dense dermal and epidermal infiltrate of CD8+ GNLY+ cells in close contact with nevus cells (Fig. 3c). These double-positive cells were completely absent in healthy skin (Fig. 3e). CD56+ GNLY+ NK cells were also upregulated in lesions of vitiligo and halo nevus (Fig. 3h), although, they predominated in the lesions of halo nevus compared to vitiligo. CD56+ GNLY+ NK cells were found in dermal infiltrates in vitiligo (Fig. 3b) and in the vicinity of nevus cells (Fig. 3d). In contrast, there were no CD56+ GNLY+ NK cells in healthy skin (Fig. 3f). It is noteworthy that almost all CD8+ cells in vitiligo lesions and to a lesser extent in halo nevus lesions expressed GNLY+ (Fig. 3g). This is in contrast to the CD56+ NK cells, where fewer cells were GNLY+ and which predominated in halo nevus lesions compared to vitiligo (Fig. 3h).

### CD8+ T lymphocytes expressing granulysin are increased in the perilesional skin of vitiligo and halo nevus

**Figure 4:**
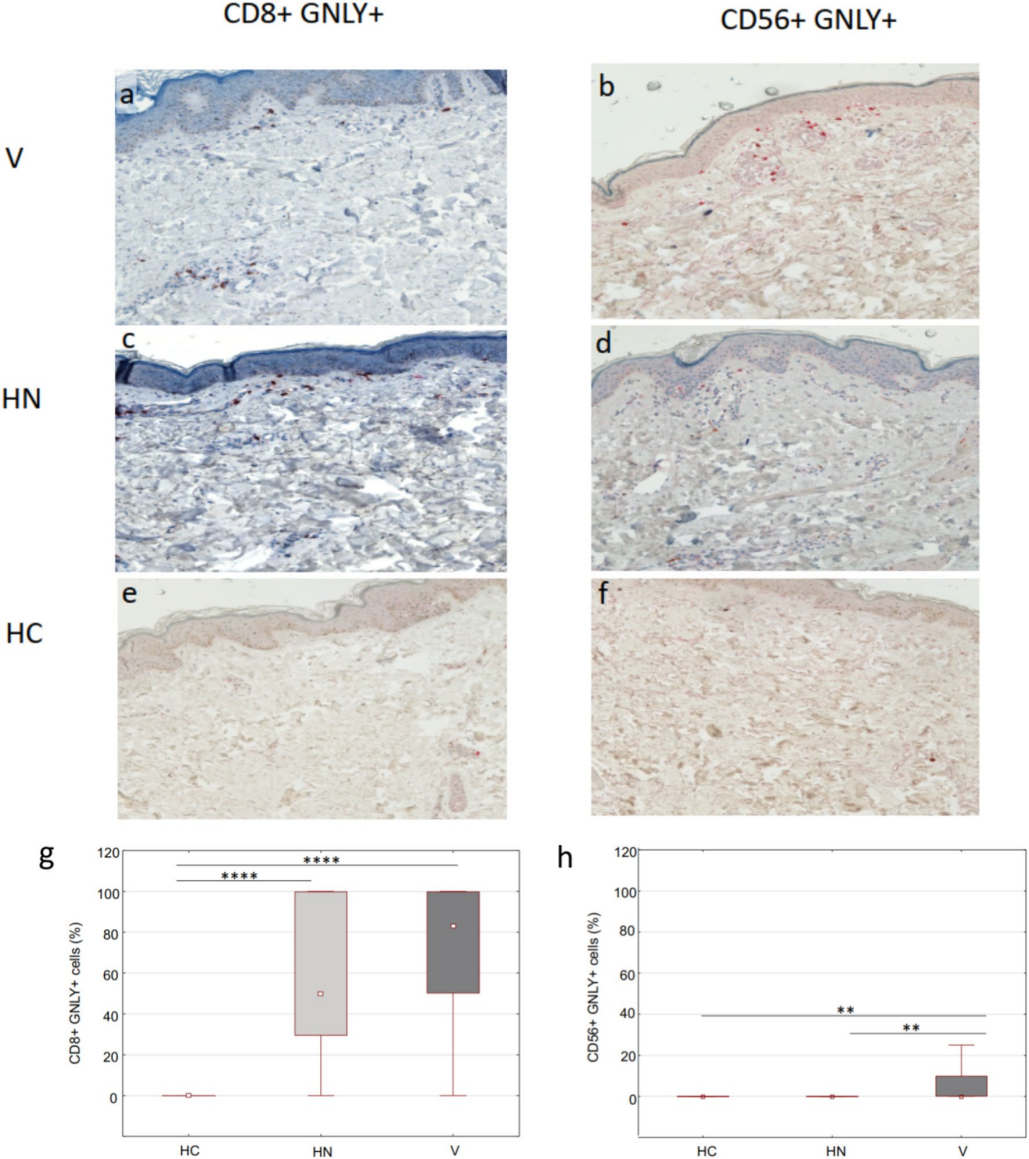
Immunohistochemical evaluation of double­positive GNLY+ CD8+ and GNLY+ CD56+ cells in perilesional skin of vitiligo and halo nevus compared to HC (**a**–**f**; magnification × 200). Surface markers CD8 and CD56 are stained red and GNLY is stained brown. Double-positive CD8+ GNLY+ cells and CD56+ GNLY+ cells are stained with both colours. Quantitative analysis of double­positive GNLY+ CD8+ and GNLY+ CD56+ cells in perilesional skin of vitiligo (n = 10) and halo nevus (n = 8) compared to HC (n = 10) (**g**, **h**). Results are expressed as the median value-25th/75th percentile. Level of significance: ****p < 0.0001, ***p < 0.001, **p < 0.01. *HC* healthy controls, *V* vitiligo, *HN* halo nevus.

A significantly higher accumulation of CD8+ GNLY+ cells was found in the perilesional skin of vitiligo and halo nevus compared to healthy skin (Fig. 4g). In vitiligo, most CD8+ GNLY+ cells were located in the perilesional dermis near melanocytes (Fig. 4a), whereas they were completely absent in healthy skin (Fig. 4e). In halo nevus, dense infiltrates of CD8+ GNLY+ cells were accumulated in the perilesional epidermis and dermis (Fig. 4c), whereas no double-positive cells were detected in healthy skin (Fig. 4e). Similar to lesional skin, CD56+ GNLY+ NK cells were also significantly upregulated in the perilesional skin of vitiligo, although not in the perilesional skin of halo nevus (Fig. 4h). In contrast, there were no CD56+ GNLY+ NK cells in healthy skin (Fig. 4f). CD56+ GNLY+ NK cells were mainly identified in the dermal infiltrates of the perilesional skin of vitiligo (Fig. 4b), where they were significantly more abundant than in halo nevus (Fig. 4d) or healthy skin (Fig. 4f).

**Figure 5:**
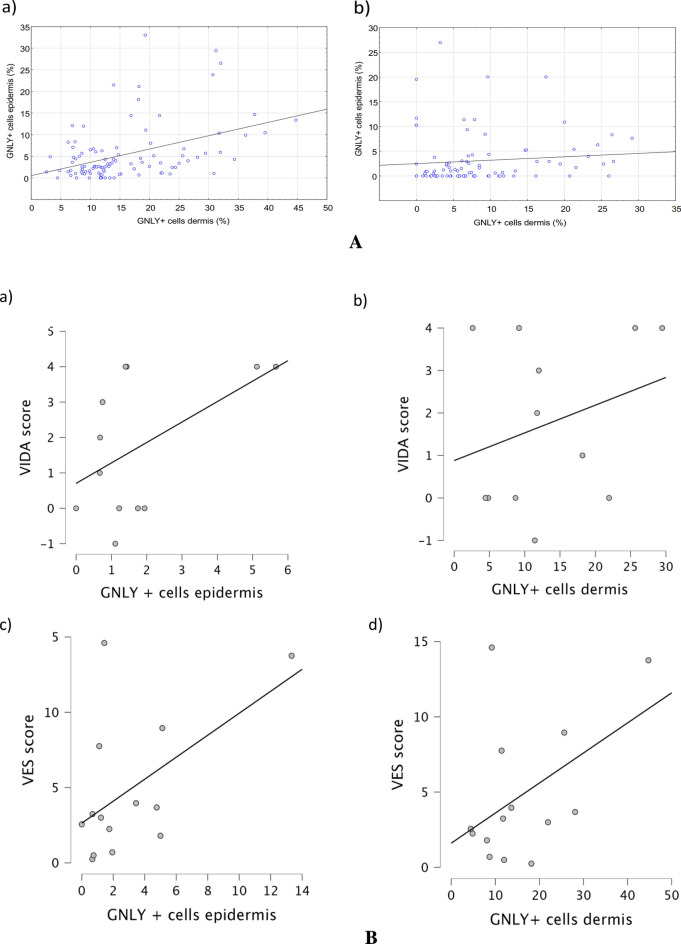
Correlation between epidermal and dermal lesional (**a**) and perilesional (**b**) GNLY in vitiligo. There was a statistically significant positive correlation between epidermal and dermal GNLY in lesional skin of vitiligo (n = 10, r = 0.346; *p* < 0.05). There was a slight, but not significant, positive correlation between epidermal and dermal GNLY in perilesional skin of vitiligo (n = 10, r = 0.191).

**Figure 6:**
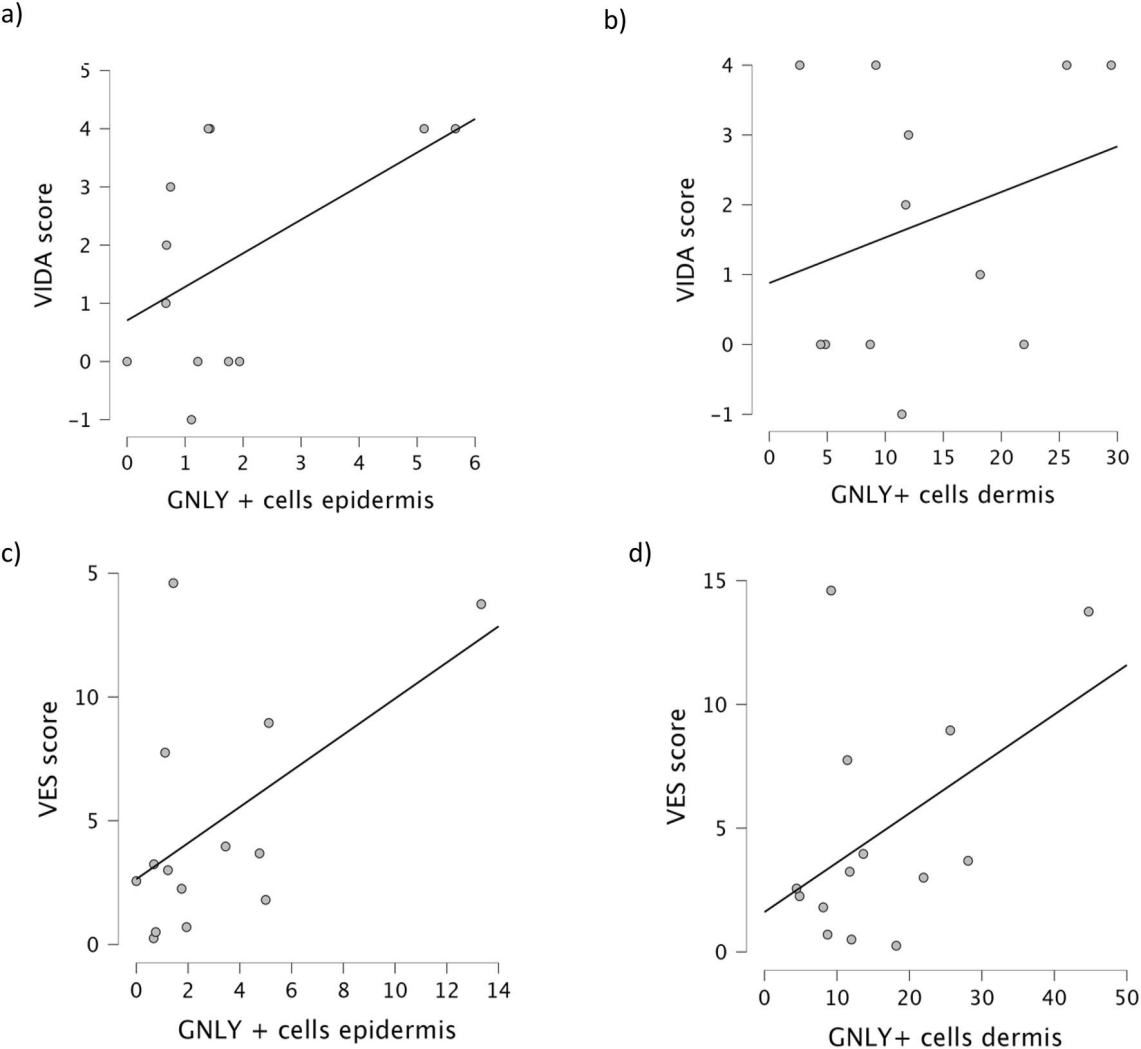
Correlation between epidermal and dermal lesional GNLY with VIDA (**a**, **b**) and VES (**c**, **d**) scores. There is a slight positive but non-significant correlation between epidermal GNLY and VIDA (n = 10, r = 0.384) or VES (n = 10, r = 0.437) scores. Similarly, there is a slight positive but non-significant correlation between dermal GNLY in vitiligo lesions and VIDA (n = 10, r = 0.268) or VES scores (n = 10, r = 0.354).

There was a statistically significant positive correlation between epidermal and dermal granulysin in the lesional skin of vitiligo (Fig. 5). There was also a positive correlation between epidermal and dermal GNLY in the perilesional skin of vitiligo, although not significant (Fig. 5). There was a slight but non-significant positive correlation between the expression of GNLY in the epidermis and dermis of vitiligo lesions and the VES or VIDA score (Fig. 6).

## Discussion

Recent studies have demonstrated that vitiligo and halo nevus are inflammatory immune-mediated disorders characterized by similar immunopathogenesis that leads to the destruction of melanocytes^3,5,34,35^. So far, it appears that CTLs, particularly CD8+ T cells, play a key role in the initiation and maintenance of both diseases^3,18,36,37^. Increased infiltration of CD8+ T cells in the lesional and perilesional skin, mainly in close proximity to neighboring melanocytes, has been observed previously and also in this study in both, vitiligo and halo nevus^3,18,38^. Recently, it has also been confirmed that after stimulation with a specific melanocyte antigen, lesional CD8+ T cells are activated, followed by upregulation of cytolytic molecules, granzyme-B and perforin^21,39^. Perforin+ and granzyme+ cells were found in tissue-resident CD8+ T cells in the epidermis and dermis of vitiligo lesions^21,40^. However, there are no data in the literature on the expression of the cytolytic molecule GNLY in the lesional and perilesional skin of vitiligo and halo nevus. In depigmented lesions and perilesional skin of vitiligo, we found for the first time, accumulation of GNLY+ cells mainly in the dermis and less in the basal and suprabasal epidermis, while in lesional skin of halo nevus GNLY+ cells were found around nests of nevus cells, suggesting the possible role of GNLY in melanocyte destruction in both diseases. As expected, GNLY+ cells accumulated more frequently in lesional than perilesional skin in both diseases, but the frequency of GNLY+ cells was twice as high in halo nevus lesions as in vitiligo lesions, suggesting a stronger cytotoxic response in halo nevus lesions. Interestingly, the percentage of double positive GNLY+ CD8+ cells in the lesions and perilesional skin of patients with vitiligo was higher than in halo nevus. This may suggest that the immune response in halo nevus is more limited to the lesions and may be broader in vitiligo, allowing depigmentation to spread to wider areas of the skin over time.

GNLY is highly expressed in numerous immune-mediated skin diseases such as lichen planus, alopecia areata and psoriasis^30–33,41^. A significantly higher GNLY level was found in the blood of patients with alopecia areata, which correlated positively with the extent of hair loss, suggesting that the GNLY serum level could be a biological marker of disease activity^32^. In addition, GNLY+ T cells were recently detected in the psoriatic plaques and peripheral blood of patients with psoriasis, and the frequency of these cells correlated with the severity of the disease^30^. In our study, however, the increased expression of GNLY in the vitiligo lesions did not correlate with the VES or VIDA score, which is in line with Saad Hassan et al. who also found no correlation between perforin expression in vitiligo and the VASI or VIDA score^21^. This could be due to the fact that the expression of GNLY is not equally present in all affected areas of skin at a given time and that VES is mainly a measure of the area of skin affected by the disease, which may increase gradually, but to a small extent during disease activity^42^. In addition, data on the relationship between serum GNLY and the activity as well as severity of vitiligo are contradictory. Some authors found no significant correlation with the severity of the disease, which is in line with our research. However, one study has found a correlation between serum GNLY and activity of vitiligo^43,44^.

GNLY has cytolytic, antimicrobial, proinflammatory, chemoattractant and tumoricidal functions^24,25,27^. Recently, GNLY has been shown to act as an immune alarmin that stimulates the immune response and induces the recruitment and activation of antigen-presenting cells, such as dendritic cells as well as CD4+, CD8+ αβ T cells, monocytes and NK cells^24^. Therefore, we can hypothesise that in immune-mediated diseases such as vitiligo and halo nevus, GNLY acts not only as a cytotoxic molecule, but also as an alarmin that attracts various immune cells and stimulates the production of numerous cytokines and chemokines to create a perfect cytokine milieu for the initiation and maintenance of the diseases.

In this study, we have shown that both CD8+ T lymphocytes and CD56+ NK cells expressing GNLY are abundant in the lesional skin of vitiligo and halo nevus, but not in healthy skin. However, in the perilesional skin of halo nevus, the major CTLs expressing GNLY are CD8+ T lymphocytes, while there are no CD56+ NK cells expressing this cytotoxic molecule. The literature reports conflicting results regarding the role of CD56+ NK cells in vitiligo, while their role in development of halo nevus has not been investigated yet^20,45,46^. NK cells represent a bridge between innate and adaptive immunity, have a strong cytotoxic effect and produce a variety of chemokines and cytokines to recruit other immune cells into the lesions^47^. Previous studies on vitiligo have not shown upregulation of NK cells in vitiligo lesions^46^. However, other authors pointed out that NK cells can be detected in non-lesional vitiligo skin, where they could initiate an immune response^20^. Nonetheless, we found sporadic infiltrates of CD56+ NK cells in lesional and perilesional skin of patients with vitiligo as well as in lesional and perilesional skin of halo nevus which has not been demonstrated before. The GNLY+ expressing NK cells were found just beneath the epidermis, near the melanocytes/nevus cells in the lesional skin of vitiligo and halo nevus as well as in the perilesional skin of vitiligo, while there were no such cells in the perilesional skin of halo nevi. In the lesions of vitiligo and halo nevus, fewer CD56+ NK cells expressing GNLY were found than CD8+ T lymphocytes expressing GNLY, which may indicate that CD8+ GNLY+ might play a dominant role in both diseases.

Limitations: The data are based on immunohistochemical analyses of single and double expression of GNLY in lymphocyte subpopulations of interest. The results lack a functional characterisation of GNLY molecule in vitiligo and halo nevus. Therefore, further studies are needed to clarify whether granulysin acts as a cytotoxic or as an immune alarmin molecule in the pathogenesis of vitiligo and halo nevus.

In this study, we have demonstrated for the first time remarkable upregulation of the cytotoxic molecule GNLY in lesions of both vitiligo and halo nevus, further confirming the similarities and associations between these two immune-mediated diseases and suggesting a potential new molecule in the pathogenesis of both diseases.

## Data Availability

The data presented in this study are available on request from the corresponding author.
